# Analysis of factors associated with postoperative systemic inflammatory response syndrome in patients with urine culture-positive stone lithotripsy

**DOI:** 10.3389/fsurg.2024.1477119

**Published:** 2024-10-10

**Authors:** Xinwei Li, Yuanpeng Zhang, Hailong Ruan, Xiaoping Zhang, Lei Liu

**Affiliations:** ^1^ Department of Urology, Union Hospital, Tongji Medical College, Huazhong University of Science and Technology, Wuhan, China; ^2^ Institute of Urology, Union Hospital, Tongji Medical College, Huazhong University of Science and Technology, Wuhan, China; ^3^ Shenzhen Huazhong University of Science and Technology Research Institute, Shenzhen, China

**Keywords:** lithotripsy, protective factor, risk factor, SIRS, urolithiasis

## Abstract

**Introduction:**

Systemic inflammatory response syndrome (SIRS) is a significant postoperative complication following lithotripsy, particularly in patients with positive urine cultures. Understanding the factors that contribute to the development of SIRS in these patients is crucial for improving clinical outcomes and reducing morbidity.

**Materials and methods:**

From 2022 to 2023, patients with preoperative positive urine culture who underwent minimally invasive uroscopic lithotripsy in Wuhan Union Hospital were retrospectively analyzed.

**Results:**

A total of 393 patients with positive urine cultures underwent endoscopic lithotripsy, and 13.2% (52/393) were diagnosed with SIRS by relevant indicators after surgery. Multivariate logistic regression was used to study the risk factors for the occurrence of SIRS in patients postoperatively, which were preoperative positive WBC in urinalysis (OR = 5.685, *p* = 0.0051) and postoperative hemoglobin drop of greater than 5 g/L (OR = 2.180, *p* = 0.0145). Notably, preoperative upper urinary tract drainage was found to be a protective factor (OR = 0.4029, *p* = 0.0302), and postoperative C-reactive protein (CRP) value (OR = 1.025, *p* < 0.0001) and procalcitonin (PCT) value (OR = 1.066, *p* < 0.0001) were predictive factors. Besides, postoperative hemoglobin drop showed a weak correlation with surgical duration (r = 0.1589, *p* = 0.0016).

**Conclusions:**

In summary, our study identifies key factors affecting the occurrence of SIRS after lithotripsy for urine culture-positive stone: preoperative positive WBC in urinalysis, postoperative hemoglobin drop, and preoperative upper urinary tract drainage. And monitoring postoperative CRP and PCT levels helps to predict SIRS.

## 1 Introduction

Urolithiasis is among the most frequently encountered urological conditions. Between 1994 and 2010, the global prevalence of urinary stone disease increased from 5.2% to 8.8% (1). Depending on the size, location, and shape, upper urinary tract stones that cannot be removed on their own (≥6 mm) require different interventions such as percutaneous nephrolithotomy (PCNL), rigid ureteroscopy (rURS), flexible ureteroscopy (fURS), and extracorporeal shock wave lithotripsy (ESWL) (2, 3).

One of the most serious conditions of postoperative infection is systemic inflammatory response syndrome (SIRS). SIRS, if not diagnosed in time, can rapidly progress to septic shock with a relatively high mortality rate. Thus, well prediction and timely treatment of SIRS are important for a better outcome (4). Relevant studies have shown that a preoperative positive urine culture is a major risk factor for postoperative infection, and treatment should be based on the results of the urine culture such as upper urinary tract drainage (5). However, for patients with positive urine culture who require surgery, the factors influencing the postoperative incidence of SIRS are still unclear.

In our study, we analyzed the key information of patients with positive urine culture includes preoperative history, preoperative laboratory examinations, operation time, operation methods, intraoperative findings, postoperative vital signs, and postoperative laboratory examinations. We found out two risk factors, one protective factor, and two predictors of SIRS after upper urinary tract stone surgery of the patients with urine culture-positive stones, which provided new insights in the prevention and prediction of SIRS after infection stone lithotripsy.

## 2 Materials and methods

### 2.1 Patient data acquisition

From January 2022 to December 2023, patients at Wuhan Union Hospital with a preoperative positive urine culture who underwent minimally invasive uroscopic lithotripsy were retrospectively analyzed. The exclusion criteria were (1) a single surgery utilizing two or more types of minimally invasive uroscopic lithotripsy and (2) missing relevant data. The study was approved by the Institutional Ethics Committee of Wuhan Union Hospital.

### 2.2 Diagnostic standard

Patients were categorized into two groups according to whether they developed SIRS after surgery or not. SIRS was defined as the presence of any two or more of the following four criteria: (1) temperature >38°C or <36°C; (2) heart rate >90 /min; (3) respiratory rate >20 /min or PaCO_2_ < 32 mmHg (4.3 kPa); (4) white blood cell count >12*10^9 ^/L or <4*10^9 ^/L or >10% immature bands (4).

### 2.3 Statistical analysis

Continuous variables that were not normally distributed with heterogeneous variance were expressed as median (IQR) and compared using the Mann-Whitney test and Kruskal-Wallis test. Categorical variables were compared using the *χ*^2^ test or Fisher exact test. Variables selected in multivariate logistic regression analysis were based on past findings and clinical experience. Correlation analysis of continuous variables that were not normally distributed with heterogeneous variance was performed using Spearman's Rank Correlation Analysis. In all the analyses, *p* < 0.05 was considered statistically significant.

## 3 Results

### 3.1 Basic information of the included patients

A total of 393 patients with preoperative positive urine culture were eligible for our study, while 52 (13.23%) of them developed SIRS postoperatively. The incidence rates for PCNL, rURS, and fURS were 14.74%, 8.42%, and 14.82%, respectively. Among all characteristics, surgical duration, preoperative WBC in urinalysis, preoperative nitrites in urinalysis, and hemoglobin drop showed significant differences between the SIRS and non-SIRS group. Their detailed information was shown in Table 1.

**Table 1:** Participant characteristics.

	SIRS (*n* = 52)	Non-SIRS (*n* = 341)	*p* value
Surgical approach, *n* (%)			0.2829
PCNL	28 (53.8)	162 (47.5)	
rURS	8 (15.4)	87 (25.5)	
fURS	16 (30.8)	92 (27.0)	
Sex, *n* (%)			0.1675
Male	17 (32.7)	146 (42.8)	
Female	35 (67.3)	195 (57.2)	
Age, median (IQR), years	53.5 (47.0–60.0)	56.0 (47.0–62.5)	0.1336
BMI, median (IQR), kg/m^2^	23.4 (21.8–26.2)	23.7 (21.6–26.3)	0.6480
Diabetes, *n* (%)			0.7772
Yes	7 (13.5)	51 (15.0)	
No	45 (86.5)	290 (85.0)	
History of fever, *n* (%)			0.2699
Yes	10 (19.2)	46 (13.5)	
No	42 (80.8)	295 (86.5)	
Preoperative upper urinary tract drainage, *n* (%)			0.2145
Yes	9 (17.3)	86 (25.2)	
No	43 (82.7)	255 (74.8)	
Preoperative use of sensitive antibiotics or intraoperative use of restricted-level antibiotics, *n* (%)			0.4096
Yes	36 (69.2)	216 (63.3)	
No	16 (30.8)	125 (36.7)	
Surgical duration, median (IQR), min	92.5 (63.5–126.8)	70.0 (50.0–110.0)	0.0092
Intraoperative signs of infection^a^, *n* (%)			0.5535
Yes	25 (48.1)	149 (43.7)	
No	27 (51.9)	192 (56.3)	
Preoperative WBC in urinalysis, *n* (%)			0.0045
Positive	49 (94.2)	263 (77.1)	
Negative	3 (5.8)	78 (22.9)	
Preoperative nitrites in urinalysis, *n* (%)			0.0032
Positive	25 (48.1)	95 (27.9)	
Negative	27 (51.9)	246 (72.1)	
Preoperative WBC count in complete blood count, median (IQR), *10^9 ^/L	6.3 (5.0–7.4)	5.9 (4.9–7.5)	0.4280
Hemoglobin drop, median (IQR), g/L	8.5 (1.3–19.5)	5.0 (0.0–11.0)	0.0348

^a^ Pus, flocculent debris, and cloudy urine.

### 3.2 Risk factors and protective factors

Based on previous studies and clinical experience, the following were selected for multivariate logistic regression analysis: history of fever, preoperative upper urinary tract drainage, preoperative use of sensitive antibiotics or intraoperative use of restricted-level antibiotics (such as vancomycin, meropenem, imipenem, teicoplanin, linezolid, and tigecycline), preoperative positive WBC in urinalysis, and a hemoglobin drop exceeding 5 g/L (3, 4). The variables included will ensure independence from each other so that there is no correlation between the two variables. For instance, preoperative WBC in urinalysis and preoperative nitrites may have a strong correlation, we only selected preoperative WBC in urinalysis as one of the regression variables. Furthermore, considering that the factors affecting the surgical duration are multifactorial, related to the location and size of the stone, the power of the laser, etc. Besides, the prolonged surgical duration brings uncertainty in the results. For example, prolonged surgical duration resulting from severe obstruction is a risk factor for SIRS, whereas prolonged surgical duration due to carefully operating is a protective factor. Therefore, we did not include the surgical duration in the regression analysis. The results of multivariate logistic regression analysis were presented in Table 2. Risk factors of postoperative SIRS include preoperative WBC in urinalysis and hemoglobin drop exceeds 5 g/L, while protective factor contains preoperative upper urinary tract drainage.

**Table 2:** Multivariate logistic regression analysis of SIRS (risk factors and protective factors).

	OR	95%CI	*p* value
History of fever	1.736	0.7337–3.889	0.1905
Preoperative upper urinary tract drainage	0.4029	0.1673–0.8788	0.0302
Preoperative use of sensitive antibiotics or intraoperative use of restricted-level antibiotics	1.076	0.5646–2.119	0.8273
Preoperative positive WBC in urinalysis	5.685	1.956–24.19	0.0051
Hemoglobin drop exceeds 5 g/L	2.180	1.181–4.145	0.0145

### 3.3 Predictive factors

Similarly, postoperative C-reactive protein (CRP), procalcitonin (PCT), and serum creatinine were included in the multivariate logistic regression analysis. The results of multivariate logistic regression analysis were presented in Table 3. It showed that postoperative CRP and PCT could be effective predictors of postoperative SIRS.

**Table 3:** Multivariate logistic regression analysis of SIRS (predictive factors).

	OR	95%CI	*p* value
CRP	1.025	1.015–1.036	<0.0001
PCT	1.066	1.035–1.101	<0.0001
Scr	0.9970	0.9892–1.003	0.3885

### 3.4 Factors affecting the decrease in hemoglobin

Kruskal–Wallis test was performed on the postoperative hemoglobin drop of all three surgical procedures PCNL, rURS, and fURS, while the results showed no statistically significant difference (H = 2.879, *p* = 0.2371). However, the correlation analysis between surgical duration and the decrease in hemoglobin showed a weak correlation with statistically significant difference (r = 0.1589, *p* = 0.0016).

As for PCNL, the size of the nephrostomy sheath did not have a direct impact on the operative time (r = 0.1058, *p* = 0.1462) and the hemoglobin drop (r = 0.1229, *p* = 0.0913), while the use of EMS could effectively shorten the operative time (U = 1,080, *p* = 0.0062) without significant hemoglobin drop (U = 2,160, *p* = 0.1338).

## 4 Discussion

SIRS, which lacks a specific treatment regimen, marks the early stage of urosepsis and often persists throughout its progression, posing significant risks to both morbidity and mortality (3). Similar to previous studies, our data also identified several factors associated with SIRS after lithotripsy for infectious urinary stones. These include risk factors such as preoperative WBC in urinalysis, protective factors such as preoperative upper urinary tract drainage, and predictive factors such as CRP and PCT in the postoperative complete blood count (5–7). In addition, relevant studies have shown that people with positive preoperative nitrites on are more likely to develop septic shock (8). However, since positive leukocytes in the urinalysis were included in the multivariate logistic regression analysis, positive nitrites, which correlate with positive leukocytes, were excluded to maintain variable independence.

Interestingly, to date, no literature has suggested an association between postoperative hemoglobin drop and SIRS, which our multivariate logistic regression analysis identified. The result may be attributed to intraoperative vascular wall injury, which subsequently leads to the entry of infectious bacteria attached to stone particles into the bloodstream. However, this also awaits a clearer causality and needs more detailed evidence, as sometimes severe infections can also lead to a hemoglobin drop. We further analyzed the potentially influential factors for the decrease in postoperative hemoglobin and found no significant differences among the three surgical approaches. However, the surgical duration showed a weak correlation with the decrease in postoperative hemoglobin. This suggests that while the correlation between these two variables is not strong, an underlying association might exist that requires a larger sample size and more precise data for accurate measurement. Regarding PCNL, we found no statistically significant difference in the impact of nephrostomy sheath size on hemoglobin drop. Besides, whether or not using EMS had no significant effect on hemoglobin drop, but the use of EMS could effectively shorten the operative time. This may be due to the higher energy level of EMS, which accelerate the surgery but not causing more injury of kidney (9).

Although our data found a slightly higher incidence of SIRS after PCNL and fURS than rURS, the difference was not statistically significant. And the coverage of PCNL and fURS, which mainly target renal stones and some upper ureteral stones, does not overlap with that of rURS. The factors most closely associated with the occurrence of SIRS were preoperative positive urine WBC and postoperative hemoglobin drop. Besides, the preoperative placement of a double J-stent or nephrostomy tube could reduce the incidence of SIRS. Perhaps in the case of patients with infectious stones, whose urinalysis suggests a large number of leukocytes and CT suggests severe obstruction, drainage should be performed in advance in all patients. And more attention should be paid to minimize vascular wall injury to reduce the possibility of SIRS. In addition, the patient's CRP and PCT should be monitored in the postoperative period, which are good predictors of SIRS. Combining preoperative and intraoperative conditions, if they are abnormally elevated in the 12 h after the surgery or even earlier, it is necessary to choose sensitive antibiotics in a timely manner to prevent septic shock. Furthermore, for larger stones requiring longer surgical duration, the use of EMS can effectively shorten the operative time and improve the stone clearance rate (9, 10). And the 22Fr nephrostomy sheath that is suitable for EMS wouldn't cause an extra hemoglobin drop.

However, our study does have some limitations. Our sample size is still relatively small and lacks more precise data. The number of people who developed SIRS after all three surgical procedures was small, especially after rURS, and the number of people who developed SIRS after rURS was less than 10. Therefore, performing multivariate regression analysis of the risk factors for SIRS in the subgroups of the different surgical procedures would result in statistical error. Additionally, there are certain influential factors that we were unable to consider, such as the surgeon's experience, intrarenal pressure, stone location, severity of hydronephrosis, and duration of obstruction.

In a nutshell, our study has identified several factors associated with SIRS following lithotripsy for infectious urinary stones. Preoperative positive WBC in urinalysis and postoperative hemoglobin drop were found to be significantly associated with SIRS. Preoperative upper urinary tract drainage appears to mitigate the risk of SIRS. Monitoring postoperative CRP and PCT levels can help predict the occurrence of SIRS. Future studies with larger cohorts and comprehensive data collection are needed to validate these results and explore other potential influencing factors. Overall, our findings suggest strategies for reducing the risk of SIRS and improving outcomes in patients undergoing lithotripsy for infectious urinary stones.

## Data Availability

The raw data supporting the conclusions of this article will be made available by the authors, without undue reservation.
